# High spatial and temporal resolution cerebrovascular reactivity for humans and large mammals: A technological description of integrated fNIRS and niABP mapping system

**DOI:** 10.3389/fphys.2023.1124268

**Published:** 2023-01-23

**Authors:** Amanjyot Singh Sainbhi, Logan Froese, Alwyn Gomez, Izzy Marquez, Fiorella Amenta, Carleen Batson, Kevin Y. Stein, Frederick A. Zeiler

**Affiliations:** ^1^ Biomedical Engineering, Price Faculty of Engineering, University of Manitoba, Winnipeg, MB, Canada; ^2^ Section of Neurosurgery, Department of Surgery, Rady Faculty of Health Sciences, University of Manitoba, Winnipeg, MB, Canada; ^3^ Department of Human Anatomy and Cell Science, Rady Faculty of Health Sciences, University of Manitoba, Winnipeg, MB, Canada; ^4^ Undergraduate Engineering Program, Department of Biosystems Engineering, Price Faculty of Engineering, University of Manitoba, Winnipeg, MB, Canada; ^5^ Department of Clinical Neuroscience, Karolinska Institutet, Stockholm, Sweden; ^6^ Division of Anaesthesia, Department of Medicine, Addenbrooke’s Hospital, University of Cambridge, Cambridge, United Kingdom

**Keywords:** cerebrovascular reactivity mapping system, cerebral autoregulation, near-infrared spectroscopy, neuroimaging system, cerebral heat maps, high temporal resolution, high spatial resolution, NIRS-based indices

## Abstract

**Introduction:** The process of cerebral vessels maintaining cerebral blood flow (CBF) fairly constant over a wide range of arterial blood pressure is referred to as cerebral autoregulation (CA). Cerebrovascular reactivity is the mechanism behind this process, which maintains CBF through constriction and dilation of cerebral vessels. Traditionally CA has been assessed statistically, limited by large, immobile, and costly neuroimaging platforms. However, with recent technology advancement, dynamic autoregulation assessment is able to provide more detailed information on the evolution of CA over long periods of time with continuous assessment. Yet, to date, such continuous assessments have been hampered by low temporal and spatial resolution systems, that are typically reliant on invasive point estimations of pulsatile CBF or cerebral blood volume using commercially available technology.

**Methods:** Using a combination of multi-channel functional near-infrared spectroscopy and non-invasive arterial blood pressure devices, we were able to create a system that visualizes CA metrics by converting them to heat maps drawn on a template of human brain.

**Results:** The custom Python heat map module works in “offline” mode to visually portray the CA index per channel with the use of colourmap. The module was tested on two different mapping grids, 8 channel and 24 channel, using data from two separate recordings and the Python heat map module was able read the CA indices file and represent the data visually at a preselected rate of 10 s.

**Conclusion:** The generation of the heat maps are entirely non-invasive, with high temporal and spatial resolution by leveraging the recent advances in NIRS technology along with niABP. The CA mapping system is in its initial stage and development plans are ready to transform it from “offline” to real-time heat map generation.

## Introduction

The concept of cerebral autoregulation (CA) refers to the cerebral vessels being able to maintain cerebral blood flow (CBF), relatively constant, over a wide range of arterial blood pressure (ABP) (Fog, 1938; Lassen, 1959). The mechanism behind this process is known as cerebrovascular reactivity (CVR) where constant blood flow is maintained through the constriction and dilation of cerebral vessels. CA can be visually represented by the Lassen autoregulatory curve, which plots CBF against cerebral perfusion pressure (CPP) or mean arterial pressure (MAP) (Lassen, 1974). This curve depicts relatively constant CBF between the lower and upper limits of autoregulation (LLA and ULA). The LLA and ULA are important and if CPP/MAP moves below the LLA or above the ULA then the autoregulatory mechanisms are unable to maintain the CBF, hence exposing the brain to pressure-passive flow states of hypoperfusion (i.e., ischemia) or hyperperfusion (i.e., hyperemia), respectively (Fog, 1938; Lassen, 1959; 1974). CA impairment manifests by change in positions of the autoregulation limit on the Lassen autoregulatory curve or, in the worst of cases, a complete absence of this curve (Budohoski et al., 2013; Zeiler et al., 2020b). Such impairments have been documented in various neuropathological states (Budohoski et al., 2013; Xiong et al., 2017; Budohoski and Czosnyka, 2018; Czosnyka et al., 2020), including traumatic brain injury (TBI) (Czosnyka et al., 1997; Sorrentino et al., 2012; Donnelly et al., 2019; Zeiler et al., 2020a; Bennis et al., 2020; Åkerlund et al., 2020; Depreitere et al., 2021). Recent literature suggests that leaving the brain exposed to the burden of impaired CA is a significant driver of poor long-term outcomes in various neurological conditions (Güiza et al., 2015; 2017; Donnelly et al., 2019; 2021; Zeiler et al., 2020a; Åkerlund et al., 2020), which makes it crucial to monitor CA continuously and accurately at the bedside.

Autoregulation can be assessed by two different types of techniques termed static and dynamic autoregulation. The main difference between them is the time scale where static autoregulation looks at changes from minutes to hours while dynamic autoregulation examines changes from seconds to minutes. Both techniques assess autoregulation by looking at pulsatile cerebral blood volume (CBV) or CBF and changes in driving pressure, however, static autoregulation looks at these measures after they have reached steady state while dynamic autoregulation assesses CA during rapid manipulation of CPP/MAP or spontaneous oscillations of CPP/MAP (Tiecks et al., 1995; Purkayastha and Sorond, 2012; Sainbhi et al., 2022). It is important to note that these two techniques do not define their temporality, so static vs. dynamic does not refer to the technique being continuous vs. intermittent.

Continuous vs. intermittent techniques point to the temporal resolution of the CA measurement, where continuous refers to regularly updating measures and intermittent refers to a single momentary measure at a point in time (i.e., snapshot). Traditionally, CA has been assessed intermittently but with the advancement of technology, continuous measures of CA are becoming more popular since they can provide more detail on the evolution of CA over long periods of time. Recently, we have conducted a narrative review on various non-invasive and minimally-invasive modalities which assess CA in an intermittent, semi-intermittent, or continuous manner (Sainbhi et al., 2022), and we refer the interested reader to this piece for more details and various methodologies of CA measurement.

Continuous CA indices are termed CVR metrics since they have not been fully validated as measures of the Lassen autoregulatory curve. These CVR metrics evaluate the relationship between slow vasogenic fluctuations in CPP/MAP and a surrogate for pulsatile CBV or CBF. Invasive, minimally-invasive, and non-invasive modalities can be used to obtain raw continuous physiological signals and derive the surrogate measures for pulsatile CBV/CBF. Such invasive modalities include intracranial pressure (ICP) (Czosnyka et al., 1997; Jaeger et al., 2006; Zweifel et al., 2008; Sorrentino et al., 2012), brain tissue oxygen (PbtO_2_) (Jaeger et al., 2006; Dengler et al., 2013), thermal diffusion flowmetry (TDF) (Rosenthal et al., 2011; Dias et al., 2015; Highton et al., 2015), and laser Doppler flowmetry (LDF) (Brady et al., 2008; Zweifel et al., 2010c; Lee et al., 2011; 2012; Zeiler et al., 2018a; 2018c). The minimally-invasive modalities include magnetic resonance imaging (MRI) (Saeed et al., 2011), positron emission tomography (PET) (Steiner L. et al., 2003; 2003a; 2003b; Coles et al., 2004; Zweifel et al., 2008), and computed tomography (CT) (Wintermark et al., 2006; Chieregato et al., 2007; Peterson and Chesnut, 2009) while the non-invasive modalities include transcranial Doppler (TCD) (Czosnyka et al., 1997; 2003; Schmidt et al., 2003) and near-infrared spectroscopy (NIRS) (Zweifel et al., 2010b; Diedler et al., 2011; Zeiler et al., 2017a; Chen et al., 2020; Sainbhi et al., 2022). CVR metrics based in time-domain from various modalities have seen widespread adoption by clinicians at bedside due to their simple, natural interpretation over the frequency-domain metrics. Preclinical models are scarce to validate these measures against the Lassen autoregulatory curve. A recent systematically conducted scoping review from our group has demonstrated that most measured indices, namely ICP and NIRS based metrics, were able to accurately distinguish CPP/MAP from above and below the LLA in pre-clinical models (Sainbhi et al., 2021). However, none of the studies were able to assess the ULA due to cardiac failure at higher CPP/MAP in these animal models (Sainbhi et al., 2021).

Currently the most established method for the continuous bedside assessment of CVR is the pressure reactivity index (PRx, correlation between ICP and MAP) since it has been validated to accurately detect the LLA in animal models (Zweifel et al., 2008; Lee et al., 2009). The ICP-based indices are limited by their spatial resolution and their requirement of invasively monitoring ICP that requires neurosurgical or neurocritical care expertise only available in specialist centres. Recently, NIRS-based indices have been described, leveraging the non-invasive nature of commercially available cerebral oximetry systems, and they can be used as a substitute for ICP-based indices (Lee et al., 2009; Zweifel et al., 2010b; Smielewski et al., 2010). Dr. Lee and others have shown that Total Hemoglobin Index (tHbx), the moving linear correlation between slow waves of total hemoglobin (tHb), correlates with PRx (r = 0.73) in animals (Lee et al., 2009). In 32 human TBI patient data, tHbx has also shown a significant correlation between PRx and tHbx (r = 0.65, *p* < 0.0001) (Zweifel et al., 2010b). This can overcome the invasive limitations of the standard PRx index, while providing the added advantage of evaluating hemispheric differences in CVR. However, it requires large comparative prospective trials to fully understand its role in bedside care provision (Gomez et al., 2021b).

As mentioned, a non-invasive method for the derivation of continuous CA indices in humans leverages NIRS technology. This technique uses continuous NIRS-based hemoglobin values or regional tissue oxygen saturation (rSO_2_) measures as a surrogate for pulsatile CBV (Lee et al., 2009; Brady et al., 2010; Zweifel et al., 2010b; Zeiler et al., 2017a; 2017b; Mathieu et al., 2020; Gomez et al., 2021a; Gomez and Zeiler, 2021; Zeiler, 2021). The CVR metrics are defined as moving Pearson’s correlation coefficients between slow-wave (i.e., 0.05–0.005 Hz) (Howells et al., 2015) fluctuations in a driving pressure for CBF, such as CPP/MAP, and a surrogate for pulsatile CBV/CBF, such as oxyhemoglobin (HbO), deoxyhemoglobin (HHb) or rSO_2_ (Lee et al., 2009; Brady et al., 2010; Zweifel et al., 2010b; Zeiler et al., 2017a; 2017b; Mathieu et al., 2020; Gomez et al., 2021a; Gomez and Zeiler, 2021; Zeiler, 2021). These NIRS-based indices have been validated in animal models to accurately detect the LLA (Brady et al., 2008; 2010; Lee et al., 2009; Sainbhi et al., 2021) and clinical data support associations with ICP based cerebrovascular reactivity measurement through rough estimates (Zweifel et al., 2008; 2010a; Zeiler et al., 2017a; 2017b). However, further work is required to understand the importance of NIRS-based cerebrovascular indices.

NIRS-derived CA measures using a combination of commercially available NIRS devices and non-invasive ABP (niABP) assessments have been previously described (Zweifel et al., 2010b; Gomez et al., 2021a; Gomez and Zeiler, 2021), highlighting the ability to derive continuous CA metrics in real-time at the bedside in an entirely non-invasive manner. However, multi-channel functionality is currently lacking in the commercial NIRS systems, for clinical settings, since they are limited to typically two channels (i.e., bifrontal assessments) and have a poor sampling frequency of ∼ 1Hz (Zweifel et al., 2008; 2010a; Gomez et al., 2020; 2021a). Thus, true non-invasive topographical mapping of CA function of the entire brain has been limited to snap-shot neuroimaging methods based on CT or MRI, with a lack of continuous portable systems. Such neuroimaging systems rely on expensive fixed equipment, with lengthy scan acquisition times to generate these intermittent images of CA capacity. As such, they are typically relegated to a limited number of centers, and not accessible to the wider research and clinical communities.

Thus, to improve the understanding of continuously assessed CA in humans and large mammals, a tool is needed with both improved spatial and temporal resolution which can facilitate real-time imaging of cerebrovascular function. With recent advances in NIRS technology, custom functional NIRS (fNIRS) (Reinhard et al., 2014; Chen et al., 2020; Sainbhi et al., 2022) systems are available that offer multi-channel capabilities and a sampling frequency of 250 Hz, facilitating full cerebral pulse-waveform data acquisition simultaneously from multiple brain regions. Multi-channel fNIRS systems have been used with non-invasive ABP measurement for CA assessment in the frequency domain along with visualizing CA changes (Reinhard et al., 2014; Schumacher et al., 2019; Tian et al., 2021). Building upon prior advances in the field, it is feasible to develop CA mapping system that visually display heat map signatures of time domain CA capacity while providing user-friendly customizable interface. With high temporal and spatial resolutions this will provide an entirely non-invasive bedside neuroimaging platform for cerebrovascular function in time domain that is portable and easy to use in a clinical setting.

This paper highlights the creation of a multi-channel high spatial and temporal resolution CA mapping system in time domain, using advanced fNIRS along with niABP monitor. Such work has led to a new wearable and portable imaging system which is capable of deriving CA maps of the entire brain, to visualize the derived time domain CA metrics, with high sampling rates at each point.

## Materials and methods

### Ethics

All research was conducted in accordance with local regulatory approvals, and ethics approval obtained from our University of Manitoba research ethics board (HS25527; B2022:051). All data presented was collected from the author/co-authors, obtained with fully informed consent for collection, and displayed in a de-identified manner within this manuscript. All methods were carried out in accordance with relevant guidelines and regulations, and the Declaration of Helsinki was taken into consideration and followed.

### Physiologic monitoring systems

**FIGURE 1:**
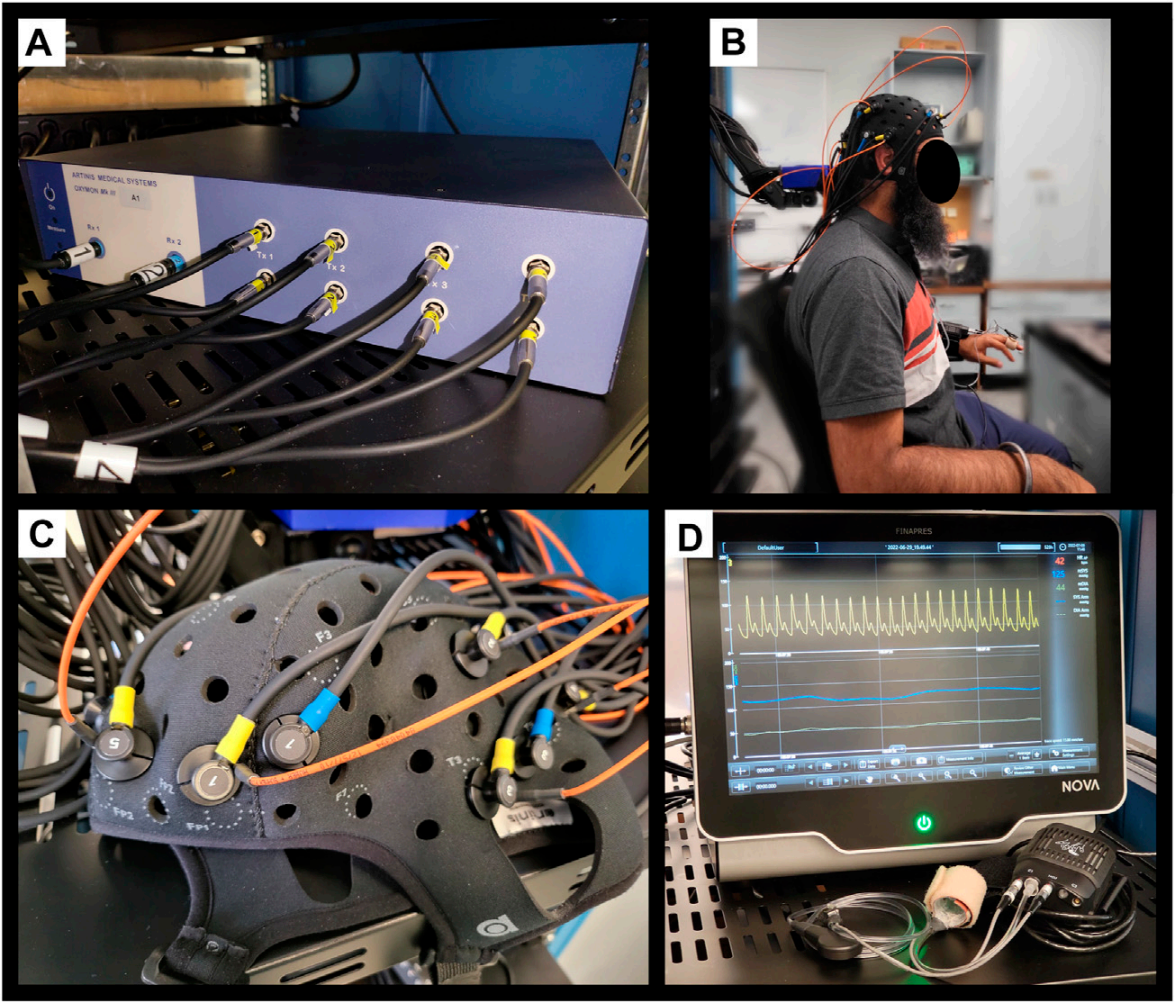
Finapres Nova and OxyMon Mk III devices **(A)**, Displays a portion of multi-channel the OxyMon Mk III device used to collect HbO, HHb, tHb, HbDiff, and rSO_2_. **(B)**, Displays the NIRS cap on human head along with niABP cuff on the finger. **(C)**, Displays transmitting optode (yellow) along with both short receiver (orange) and normal receiver (blue) for a channel on the NIRS cap in close up. **(D)**, Displays the Finapres Nova device used to collect niABP *via* finger-cuff technique. ABP, arterial blood pressure; niABP, non-invasive continuous arterial blood pressure; HbO, oxyhemoglobin; HHb, deoxyhemoglobin, tHb, total hemoglobin; HbDiff, difference between HbO and HHb; NIRS, near-infrared spectroscopy; rSO_2_, regional cerebral oxygen saturation.

**FIGURE 2:**
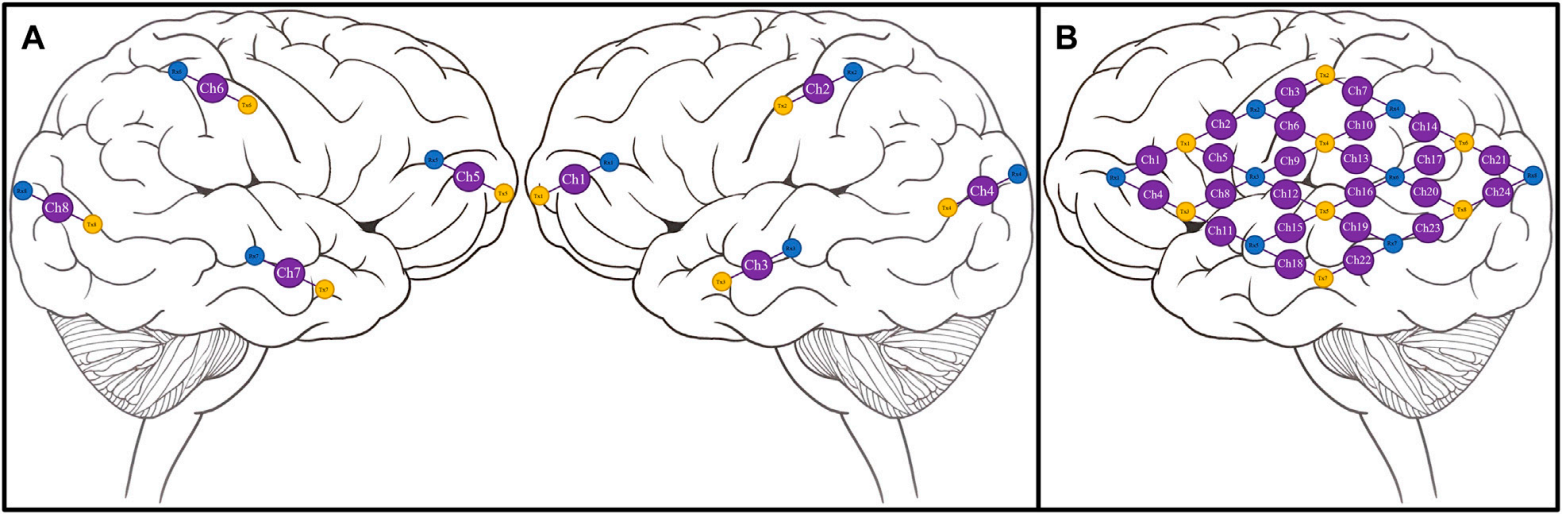
Conceptual NIRS channel layouts on a human brain template **(A)**, Conceptual 8 channel setup of NIRS device on a template of the right and left hemispheres of a human brain. **(B)**, Conceptual 24 channel grid setup of NIRS device on the left hemisphere of a human brain. Ch, channel; NIRS, near-infrared spectroscopy; Rx, receiver; Tx, transmitter. Note—grid layouts are entirely customizable for evaluating one or all regions of the brain with the required optodes, with the given rectangular grid in panel B provided as just an example.

We leveraged a custom multi-channel fNIRS system OxyMon Mk III (Artinis Medical Systems, Elst, Netherlands), as seen in Figure 1A. Equipped with eight transmitting optodes and eight receiving optodes along with an additional eight reference optodes. The system also utilizes a NIRS cap to hold various optodes in place with optode holders; Figure 1B shows the NIRS cap with optodes on a human head and an example of a receiver and transmitter optodes can be seen in Figure 1C. The combination of a transmitter and receiver optode pair creates normal channels separated by 30 mm whereas a transmitter and reference optode pair creates a short channel separated by 10 mm. The short channels are subtracted from normal channels to eliminate scalp noise and it is important to note that this system is able to eliminate scalp noise separately at each channel. This device comes with its own software called OxySoft (version 3.2.72) from where the channel layouts can be set and customized from recording with 8 separate channels, using separate receiver and transmitter optodes as seen in Figure 2A, to having a grid of multiple channels sharing receiver and transmitter optodes, as conceptualized in Figure 2B.

Next, to derive NIRS-based indices of CA, we required a continuous full-waveform ABP system, which could acquire data non-invasively and be coupled with the NIRS system described above. Thus, we utilized the continuous niABP signal from a Finapres Nova (Finapres Medical Systems, Enschede, Netherlands), as seen in Figure 1D, which uses a finger-cuff technique for full-waveform digital artery ABP acquisition and brachial artery reconstruction (using a reference brachial arm cuff). Also, Figure 1B shows the niABP finger cuff on a human finger.

### High-frequency data acquisition

**FIGURE 3:**
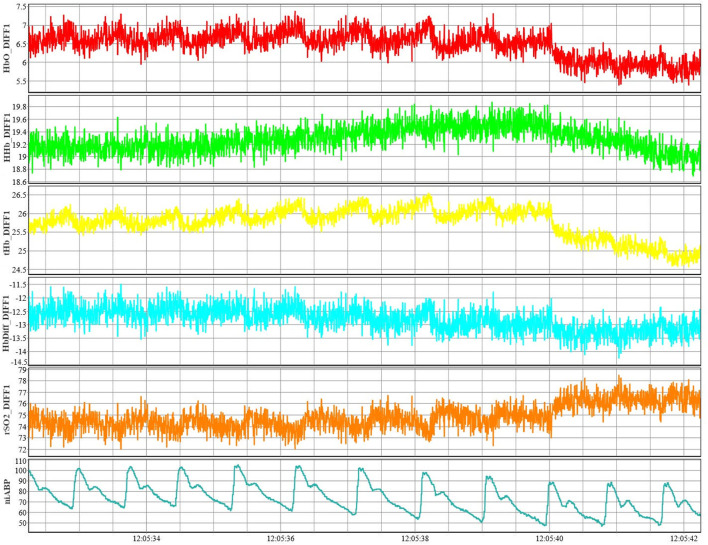
Example of NIRS signals from a single channel along with niABP signal. An example of NIRS signals of HbO, HHb, tHb, HbDiff, and rSO_2_ shown from channel 1 along with an example of niABP signal.NiABP, non-invasive continuous arterial blood pressure; HbO, oxyhemoglobin; HHb, deoxyhemoglobin, NIRS, near-infrared spectroscopy; tHb, total hemoglobin; HbDiff, difference between HbO and HHb; rSO_2_, regional cerebral oxygen saturation.

All signals are recorded in high-frequency time series using Intensive Care Monitoring “Plus” (ICM+) software (version 8.5.4.6; Cambridge Enterprise Ltd., Cambridge) connected to the data streams from the two systems. Signals from all the monitoring devices described below are recorded in time series using this software throughout the recording period. An example of these pure cerebral signals (short channel subtracted from normal channel) from a single channel are shown in Figure 3 along with the niABP signal.

The backend communications of these devices have been accomplished in our lab, facilitating success. The NIRS system uses its own software, OxySoft, to perform and view measurements with an option to output the data in real time using Lab Streaming Layer (LSL; https://github.com/sccn/labstreaminglayer). OxySoft is also able to create custom graphs by manipulating the hemoglobin values to create a new trace. For example, an rSO_2_ trace can be created as a ratio of HHb to tHb multiplied by 100 to get a percentage value. LSL system unifies collection of measurement time series, handling both networking and time-synchronization. The Finapres device outputs the niABP signal in an analogue format which is digitized using Data Translations DT9804/DT9826 converters (Data Translation, Marlboro, MA) and an LSL data stream is created using Python interface to LSL (PyLSL; https://github.com/labstreaminglayer/liblsl-Python). To record these data streams from both systems using ICM+, custom modules in Python have been developed. These modules separately read both the NIRS and Finapres streams using PyLSL and transfer the data to virtual communication (COM) ports. Then the virtual COM ports are used as input signals for the ICM + which is used for data storage. For the NIRS signal, the module is setup to add the 3.22 s delay to the NIRS LSL signal streams when transferring the data to the COM port. This delay to the NIRS signal was added to match the output signal delay of the niABP signal as measured in a series of tests whose results are shown in Supplementary Appendix SA (single run) and Supplementary Appendix SB (multiple runs).

**FIGURE 4:**
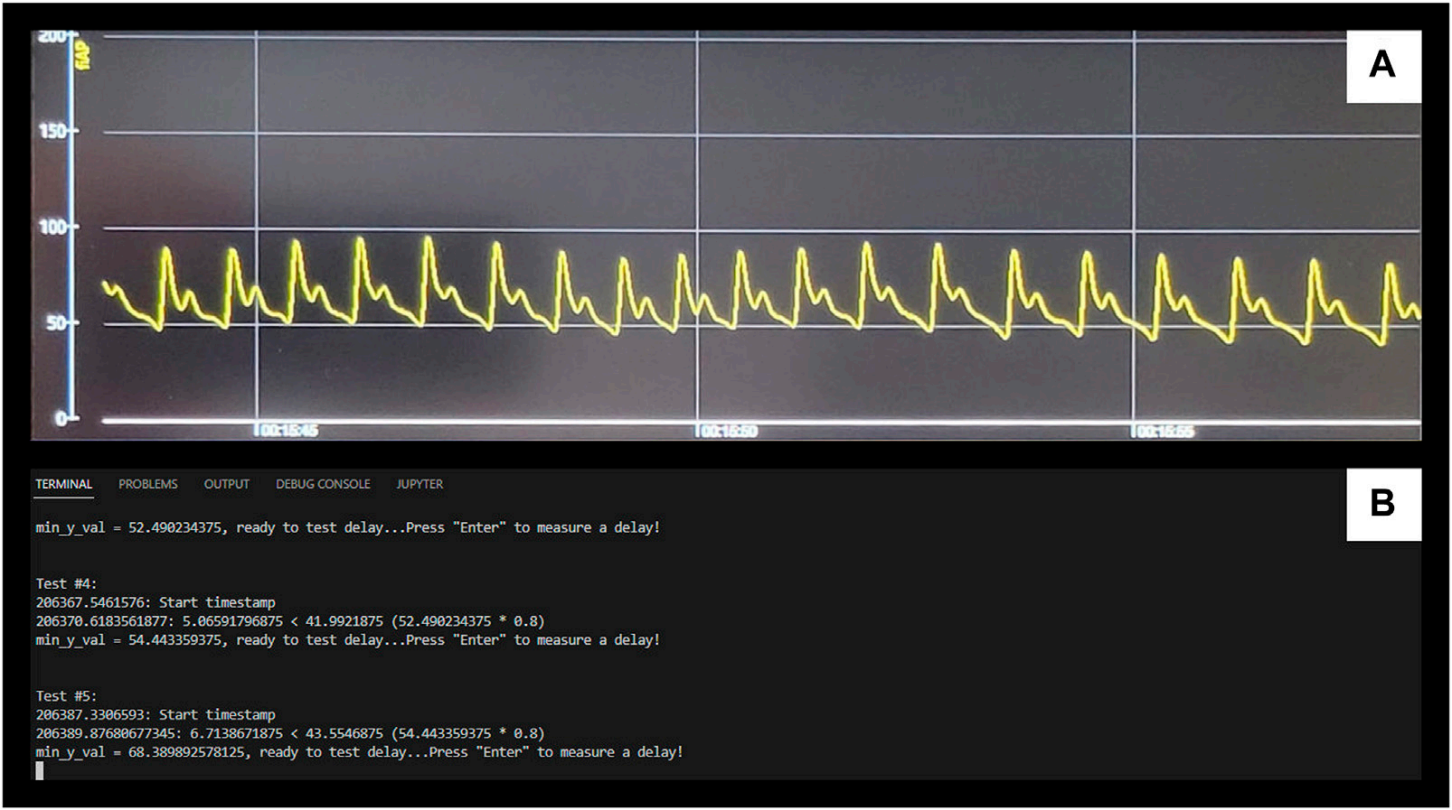
Example of Finapres Nova signal output delay test **(A)**, Displays the niABP output on the Finapres Nova display. **(B)**, Displays the output of Python module for retrieving the start and end timestamps. The drops in the signal from **(A)** correlate to “enter” being pressed on **(B)** to start a test and the end timestamp is automatically outputted when measures of the signal has dropped below a minimum value. NiABP, non-invasive continuous arterial blood pressure.

The niABP is obtained with the Finapres device at 100 Hz sampling frequency, sampled through an entirely non-invasive finger-cuff. The output signal has a delay as compared to the signal shown on the device screen. To quantify this delay, in a single run, we performed 100 tests to compare the start time of the finger-cuff disconnection to the time the signal drop showed in the output signal using pyDTOL library (https://github.com/jensb89/pyDTOL) with Python 2.7. These 100 delays, given in Supplementary Appendix SA, gave an average delay of 3.24 s. This average delay was similar to the average delay of 3.18 s found in 10 separate runs where 10 tests were conducted in each run as shown in Supplementary Appendix SB. The delay of 3.22 s was added to the NIRS signal which was calculated by averaging the average delays from Supplementary Appendix SA, SB; Figure 4 displays an example of the delay test as seen on the Finapres display (Figure 4A) and in the output of the Python module (Figure 4B).

Signals from the NIRS system at each channel are HbO, HHb, tHb, the difference between HbO and HHb (HbDiff) of regular channels, short reference channels, and the final pure cerebral signals obtained by removing scalp noise along with recording raw optical densities (OD) values. The NIRS system uses wavelengths ranging from 842–847 nm to calculate the relative HbO values and to calculate the relative HHb values, it uses wavelengths in the range of 761–762 nm. These NIRS signals are recorded using a total of eight channels with one channel per brain lobe (Frontal, Parietal, Occipital, and Temporal lobes) resulting in four channels placed on each left and right hemispheres of the brain. In addition to the eight normal channels, signals from eight reference channels are also recorded. Another example of a larger grid map has a total of 24 channels arranged in a 4 x 4 grid of receiver and transmitter optodes on the left hemisphere of the brain. The NIRS system is able to output these signals at a sampling frequency of 250 Hz.

### Physiologic data processing

At each channel, five indices are derived by first decimating the raw signals using non-overlapping moving average filters of 10-s duration which allows us to focus on the slow-wave vasogenic fluctuations associated with CA. Then Pearson correlation coefficients are calculated using 30 consecutive 10-s mean values of each NIRS signals and the niABP signal which are updated every 10-s. These five indices are Oxyhemoglobin Index (HbOx—correlation between HbO and niABP), Deoxyhemoglobin Index (HHbx—correlation between HHB and niABP), Total Hemoglobin Index (tHbx—correlation between tHb and niABP) (Lee et al., 2009; Zweifel et al., 2010b), Hemoglobin Difference Index (HbDiffx—correlation between HbDiff and niABP) which is the difference between HbO and HHb, and Cerebral Oximetry Index (COx—correlation between rSO_2_ and niABP) (Brady et al., 2010; Gomez et al., 2021a), as given in Table 1. These indices were generated using ICM + software and then outputted as comma-separated values (CSV) file.

**TABLE 1:** Derived indices at each channel.

Abbreviation	CVR metric	Correlation between
HbOx	Oxyhemoglobin Index	HbO and niABP
HHbx	Deoxyhemoglobin Index	HHb and niABP
tHbx	Total Hemoglobin Index	tHb and niABP
HbDiffx	Hemoglobin Difference Index	HbDiff and niABP
COx	Cerebral Oximetry Index	rSO_2_ and niABP

niABP, non-invasive arterial blood pressure; CVR, cerebrovascular reactivity; HbO, oxyhemoglobin; HHb, deoxyhemoglobin; tHb, total hemoglobin; HbDiff, difference between oxyhemoglobin and deoxyhemoglobin; rSO_2_, regional cerebral oxygen saturation (ratio between HHb, and tHb).

The custom multi-channel NIRS platform and the niABP systems have been integrated by developing a custom module in Python to export the HbO, HHb, Hb, and HbDiff at 250 Hz sampling frequency. Data at a matching sampling frequency is exported from Finapres NOVA device in an analogue format and digitized using Data Translation converter. This high frequency data was linked in time-series using the data-acquisition platform, ICM+, in keeping with previous work from the lab using commercial NIRS platforms (Gomez et al., 2020; 2021a). In keeping with previous work on the derivation of continuous CA indices (Zweifel et al., 2010b; Lang et al., 2015; Zeiler et al., 2017b; Gomez et al., 2021a), the raw data was decimated using non-overlapping moving average filters of 10-s duration to focus on the slow-wave vasogenic fluctuations associated with CA and filter out confounders from other frequency ranges, such as Mayer waves (Fraser et al., 2013; Howells et al., 2015). The Pearson correlation coefficients were calculated using 30 consecutive 10-s mean data from the NIRS and niABP signal and updated every 10-s. This generated five continuously updated CA indices per channel which were used as input to generate a heat map using the custom Python module developed to provide a visual representation of the CA indices data which ranges from −1.0 to +1.0.

### Heat maps

**FIGURE 5:**
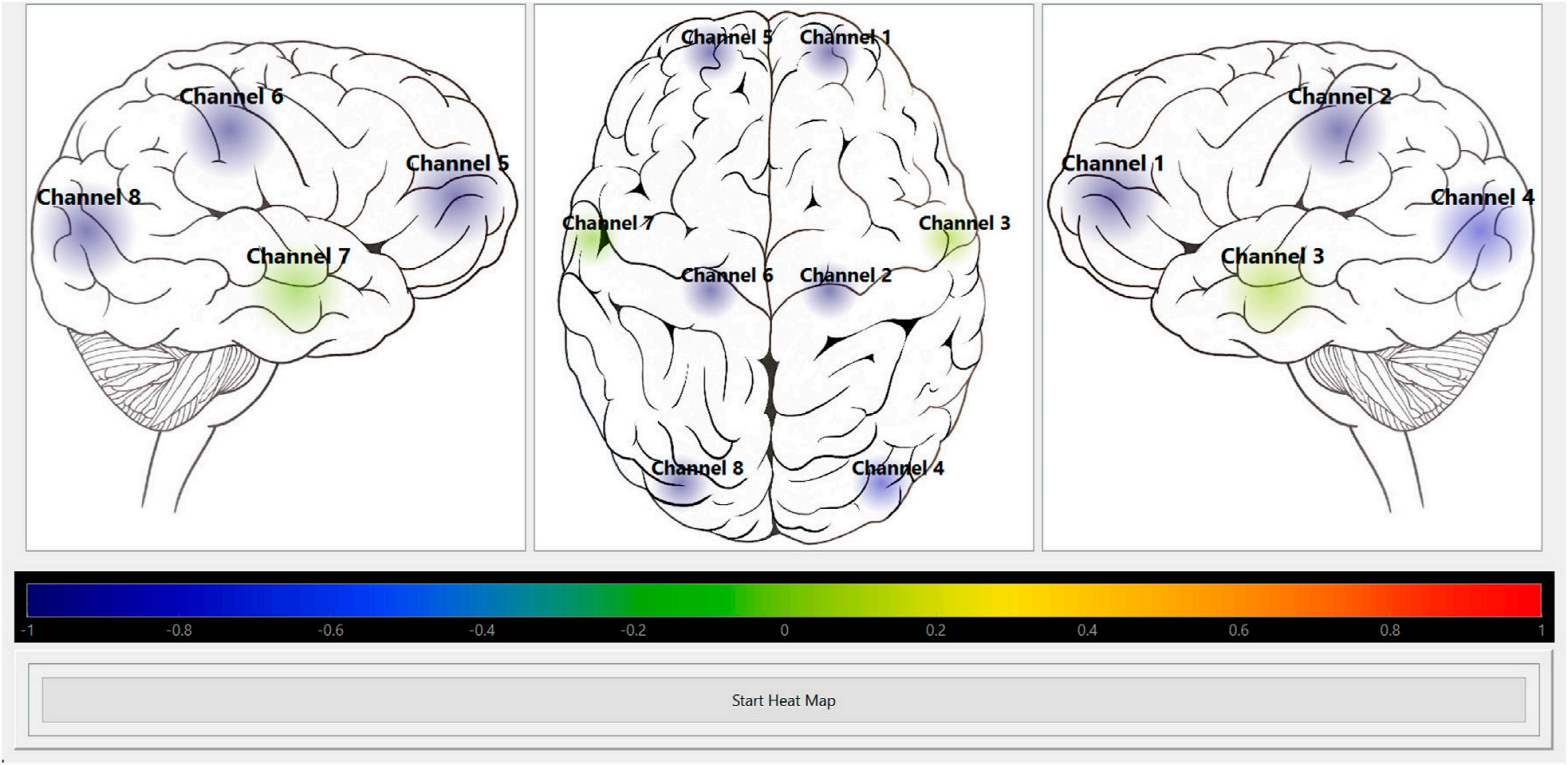
Heat map module visually displaying HbO autoregulation index for 8 channels. Shows the screenshot of the Python heat map module’s GUI running in “offline” mode for HbO autoregulation index of an 8 channel recording. The colour bar represents the CVR index scale from Blue = -1 (intact CVR) to Red = +1 (impaired CVR). CVR, cerebrovascular reactivity; GUI, graphical user interface; HbO, oxyhemoglobin.

**FIGURE 6:**
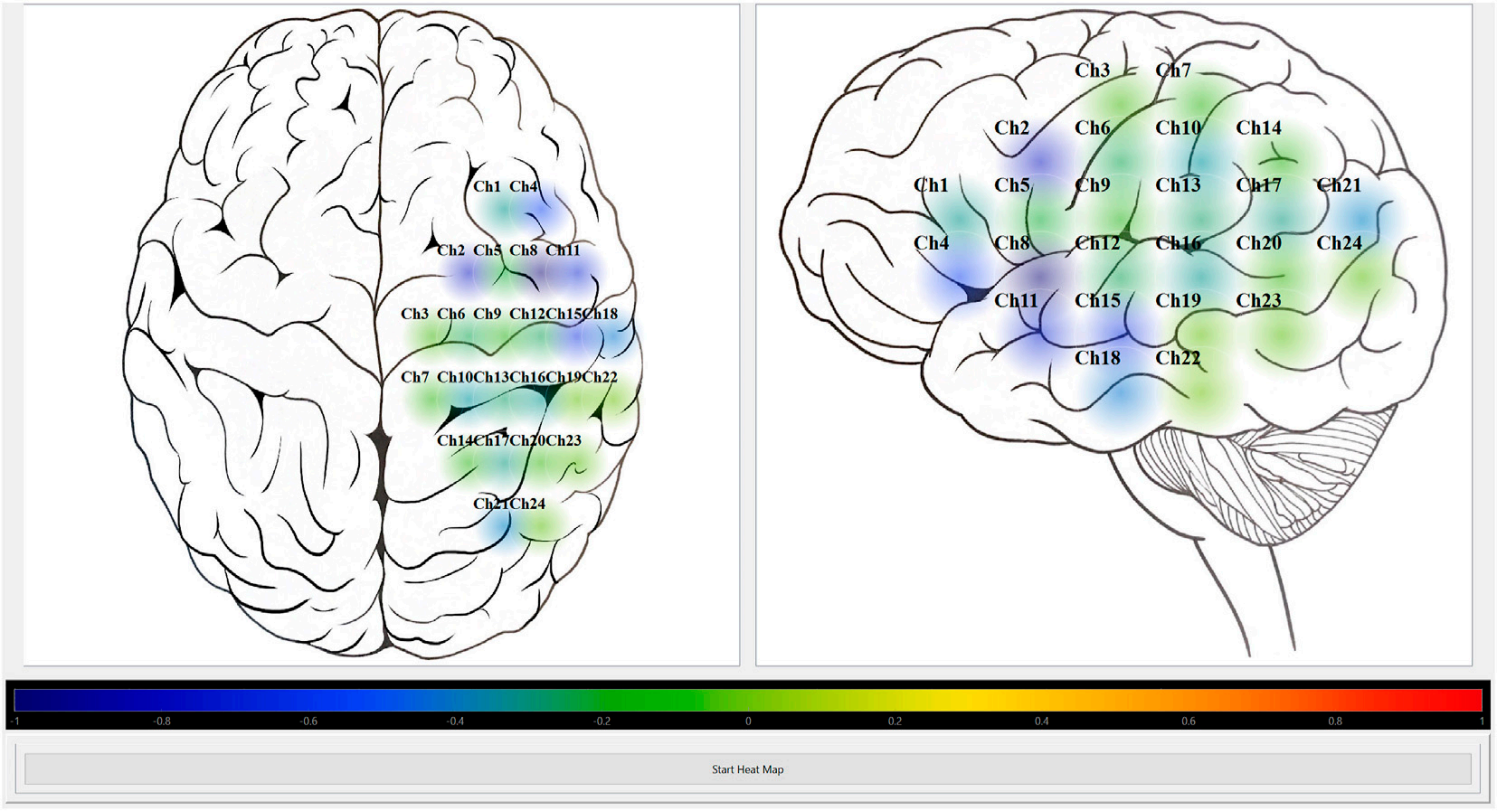
Heat map module visually displaying HbO autoregulation index for 24 channel grid. Shows the screenshot of the Python heat map module’s GUI running in “offline” mode for HbO autoregulation index of a 24 channel grid recording. The colour bar represents the CVR index scale from Blue = -1 (intact CVR) to Red = +1 (impaired CVR). CVR, cerebrovascular reactivity; GUI, graphical user interface; HbO, oxyhemoglobin.

Python module was developed to depict the calculated indices visually as a form of heat map. The current graphical user interface (GUI), as shown in Figures 5, 6, was created using Python binding, PySide6 (https://pypi.org/project/PySide6/), from the Qt for Python project (https://doc.qt.io/qtforpython/) along with PyQtGraph library (https://pyqtgraph.readthedocs.io/en/latest/). The Pandas (https://pypi.org/project/pandas/) and NumPy (https://numpy.org/doc/stable/) libraries were used to store the exported CSV file from ICM + software containing the calculated indices in a data frame and then read from it at a certain frequency (i.e., every 10-s) to update the heat map by converting the read value to a colour based on the colour map. The layouts of the optode positions relative to the 2D outline of the human brain, index type, and the rate of display were all hardcoded in the Python module. To clarify, ICM+ is used for long term data storage with the potential for “offline” heat map derivation at a later date with data stored in CSV format. The Python pipelines to read NIRS and niABP signals were mindfully developed to read data for “online” generation of updating heat maps, further discussed in Future Directions section.

## Results

The custom Python heat map module works in “offline” mode which means it can visually portray the CA index per channel with the use of colourmap. So, after saving the calculated Pearson correlation coefficients from ICM + software in a CSV file, the module is able to read that data and display it at a desired frequency.

The module was tested on two different mapping grids using data from two separate recordings. All signals were acquired and stored in ICM + then they were decimated using non-overlapping moving average filters of 10-s duration. This resulted in the creation of the five CA indices, HbOx, HHbx, tHbx, HbDiffx, and COx) (described in Table 1), that was stored as a CSV file.

The Python heat map module was able to read the CA indices from the CSV file created used ICM + software. It was successful in visually displaying a selected CA index at the preselected rate of 10-s. Figures 5, 6 show screenshots of the heat map module displaying a selected CA index, HbOx, for both 8 channel and 24 channel grid types.

The maximum number of channels the current lab setup of OxyMon with eight receivers and eight transmitters can facilitate is 48 channels at a frequency of 250 Hz although Artinis Medical Systems offer fNIRS systems with 100 + channels at 250 Hz frequency. Such frequency facilitates multi-channel acquisition of the full pulse-waveform for all fNIRS measures. It must be acknowledged, the derivation of continuous CA indices necessitates focusing on the vasogenic slow-wave frequency band, through application of low-pass filters as described in our data analysis and figures. Such CA indices are derived in a continuously updating fashion and linked in time-series with the full-waveform fNIRS and niABP data sets. This facilitates the ability to interrogate the complex relationship between CA and other metrics that are only derived from cerebral and systemic pulse-waveform data, such as pressure-flow dynamics, compliance/compensatory reserve, and autonomic function.

## Discussion

Currently, the novel mapping system is in its initial development stage. The NIRS data was recorded at 250 Hz and niABP data was recorded at 100 Hz in the ICM + software with the help of custom Python pipelines. Using ICM + software, the recorded data was decimated using non-overlapping moving average filters to focus on the slow-wave vasogenic fluctuations associated with CA and then Pearson correlation coefficients were calculated from the mean data of NIRS and niABP signals with an update frequency of 10-s, producing five continuously updated CA indices per channel.

This system relies on non-invasive devices to obtain niABP as the driving pressure for CBF, and surrogates for pulsatile CBV/CBF which are HbO, HHb, Hb, and HbDiff. By using fully non-invasive devices like NIRS and Finapres, we eliminate any risk of potential harm to the subject being evaluated. Currently, most of the existing imaging methods that assess autoregulation use either invasive or minimally-invasive devices, so leveraging the use of non-invasive devices provides a safer and more portable system to acquire the CA measurements. Compared to minimally-invasive neuroimaging based methods which employ immobile platforms such as CTP (Wintermark et al., 2004), Xe-CT (Chieregato et al., 2007), PET (Derdeyn, 2007), and MRI (Saeed et al., 2011), that assess CA statically in an intermittent fashion, NIRS assesses CA dynamically as a continuous measure, giving more data on the autoregulatory process.

The custom built NIRS device enables the customizability of the channel layouts with the eight receivers, 8 transmitters, and 8 reference receivers. So, the maximum number of channels that can be configured with the current lab setup of this device is 48 channels at a frequency of 250 Hz. Along with changing the channel configuration in the OxySoft software for the NIRS device, the channel configuration in the heat map module can also be changed with the flexibility of Python bindings from Qt for Python project. We have the freedom of adding various buttons, input fields, and log output to the heat map GUI for easy reconfiguration of properties such as channel layouts, and user-selected windows for data analysis by using Python. In the future, the number of channels can be expanded with the addition of split receiver fibers or OxyMon units.

Further, our current NIRS cap layout may be problematic for routine use within the ICU setting. The NIRS cap displayed within the current manuscript and description is the first rendition of what we expect to be many. It currently facilitates multi-channel (short and long) capacity, as well as ability for multi-channel EEG (if desired). It is acknowledged, that for regular ICU use this may not be ideal, given the presence of wounds/incisions and need for access to the scalp for drains and/or intra-cranial monitoring. As such, as we move forward, we plan to explore the development of custom “band-like” arrays that facilitate access to commonly utilized areas of the calvarium (i.e., such as Kocher’s point). Further to this, the neoprene material of the current cap is not ideal for an environment where blood products may come into contact as a result of scalp wounds or incisions. Subsequently, further cap development for ICU use will include considerations for easily cleanable materials, using accelerated hydrogen peroxide solutions, that are compliant with more widely accepted infection prevention and control measures.

Since the PRx index and NIRS-based indices are Pearson correlation coefficients, they have the same range of −1.0 to +1.0. Although the thresholds for favorable or unfavorable outcome could be different since PRx has survival threshold of 0.25 and favorable outcome threshold of 0.05 (Sorrentino et al., 2011; 2012; Zeiler et al., 2018b) while an increase of COx > 0.38 has been seen when mean velocity index (Mx), a TCD-based CA index, is above its threshold of 0.45 (Brady et al., 2010). Future healthy volunteer studies from our group will look at defining thresholds of normal and impaired CA using these NIRS-based indices.

A major asset of this system is its portability since it can be used continuously at the bedside or in out-patient clinic settings. With accurate monitoring of CA at the bedside, it can provide CA data which may help overturn the poor long-term outcomes in various neurological conditions. The ICP-based index, PRx, is the current “gold standard” for continuous bedside assessment but is limited by the invasive monitoring of ICP requiring neurosurgical or neurocritical care expertise. Since NIRS-based hemoglobin indices, such as tHbx, have previously been shown to correlate well with PRx (Lee et al., 2009; Zweifel et al., 2010b), then NIRS-based indices from our system can be used in place of ICP-based indices to overcome both the invasive and limited spatial resolution issues. Both research and clinical environments can benefit from the non-invasive nature, portability, ease of use, and relative low cost of this system. It has been found, through feasibility studies, that NIRS devices have practical use in pre-hospital setting for assessment of TBI patients as compared to the limited portability of CT and MRI machines (Weatherall et al., 2012).

## Current limitations

Presently, the system has a few limitations starting with only being able to generate cerebral heat maps in “offline” mode which requires a CSV file to be provided containing the precalculated autoregulatory indices. Secondly, the cerebral heat map display settings such as update frequency, number of optodes, and optode placement on the brain outline are hardcoded in the custom Python module without an option to adjust the settings easily with the help of GUI. Third, data acquisition modules are not integrated with the heat map module, limiting the system from being able to generate heat maps in real time during data acquisition. Fourth, each optode needs to be corrected after the NIRS cap is on a human head otherwise it affects signal quality.

## Future directions

The custom Python heat map module is at its preliminary stage of development. There are future development plans for this module. First, is to add index calculations (moving average and Pearson correlation using sliding window) as part of the module, eliminating the need to perform these calculations with another software. With these calculations, the time resolution windows will be adjustable for generation of the maps. Using Python, an autoregulatory index calculations module is in the works and it will leverage Python’s multiprocessing library to split the calculation for multiple channels amongst different processes. Also, once the module has been tested, it will get integrated into the heat map module. Second, the optode placements are currently hardcoded in the module but they will easily be adjustable using the GUI along with changing the number of optodes shown on the map. Third, the heat maps module will be integrated with the data acquisition modules to be able to generate heat maps in real time along with saving the calculated indices data to be viewed at a later time. So, this module will have “online” and “offline” modes to be able to generate the heat maps while acquiring data or using previously acquired data based on the user’s desires. Fourth, a feature of creating prediction maps for CA will be added using time-series and machine learning forecasting techniques on varying time scales. Univariate and multi-variate time-series modelling of cerebrovascular reactivity indices and niABP using Box-Jenkins time-series methodologies will be used to demonstrate the feasibility of point and interval prediction of cerebral physiologic signals by deriving autoregressive integrative moving average (ARIMA) and vector ARIMA (VARIMA) (Thelin et al., 2020; Zeiler et al., 2021). Results from this has potential to advance the application of machine learning and computational approaches to the developed novel imaging platforms that can predict cerebral physiologic responses based on past data. Integrating data from this device, with other continuous cerebral physiologic devices (i.e., ICP, PbtO_2_,and TCD) will facilitate future work on cerebral network physiologic analysis and cerebral physiologic state-space modelling, potentially with state forecasting models. Similarly, integrating cerebral physiologic data with systemic cardiovascular and autonomic data streams will enable true continuous real-time interrogation of cardio-cerebral crosstalk. Finally, physiologic data from this type of platform can facilitate integrating novel physiologic data, other big data from proteome and genome of humans and other mammals which can expand our understanding of fundamental mechanisms involved in cerebrovascular control.

Preliminary measurements have indicated that increasing the number of channels with multiple traces from OxySoft require more random accessed memory (RAM) for recording signals in ICM+. OxySoft may be using more resources than needed to calculate and update all the traces in real time which is not useful to us since we are using a different data acquisition platform, ICM+. To reduce the load on the computer, it may be beneficial to only output the hemoglobin values from OxySoft of the normal and short channels than to calculate the difference between them to get pure cerebral signals along with the rSO_2_ value using Python in the LSL to COM module. This needs more detailed investigation of the pipeline to correctly identify where more RAM is being used and if it can be reduced by offloading some calculations to a different part of the pipeline. Limiting the resources to a bare minimum during data acquisition will help free resources needed for future additions to the module such as “online” heat maps and predictive maps.

Currently there are plans to perform various perturbation challenges in healthy volunteers to create a baseline and in the future, it can be compared with patients with neurological conditions. Such work will evaluate postural change, CO_2_ challenges and neurovascular coupling (using ANAM standardized testing) in a block-trial design and has recently been supported by the Natural Sciences and Engineering Research Council of Canada (NSERC; NCT05433129).

## Conclusion

By leveraging the recent advances in NIRS technology along with niABP, we were able to create a multi-channel high spatial and temporal resolution CA mapping system. The system is in its initial stage and development plans are ready to transform this system from generating heat maps in “offline” mode to real-time heat map generation along with adding prediction capabilities using time-series and machine learning forecasting techniques. We hope it will help in improving our understanding of CA in humans and large mammals in the near future.

## Data Availability

The datasets presented in this article are not readily available because Research ethics board approval at our institution does not facilitate free and open sharing of human data, regardless of data being in a de-identified fashion. All such data is protected under both ethics and privacy acts within the Province of Manitoba, preventing such open sharing of data. All the data analyzed and used is available from the corresponding author on a reasonable request. Requests to access the datasets should be directed to AS, amanjyot.s.sainbhi@gmail.com
