# Supplementary material for: High spatial and temporal resolution cerebrovascular reactivity for humans and large mammals: A technological description of integrated fNIRS and niABP mapping system
Source: Front Physiol. 2023 Jan 23;14:1124268. doi: 10.3389/fphys.2023.1124268 (PMC9899997; doi:10.3389/fphys.2023.1124268)
Supplement: Supplementary file 1 [file Table1.docx]

**Appendix A – Finapres Signal Delay Tests (Single Run):**

| **Test** | **End Time (seconds)** | **Start Time (seconds)** | **Time Difference (seconds)** |
| --- | --- | --- | --- |
| 1 | 1422239.1374133076 | 1422235.7673302 | 3.3700831 |
| 2 | 1422259.992468897 | 1422256.7938428 | 3.19862609 |
| 3 | 1422286.0030421375 | 1422282.4566678 | 3.54637433 |
| 4 | 1422306.2694347468 | 1422303.0783761 | 3.19105864 |
| 5 | 1422331.9211692389 | 1422328.6736615 | 3.24750773 |
| 6 | 1422355.5463266862 | 1422352.4629866 | 3.08334008 |
| 7 | 1422379.5747502183 | 1422376.1509737 | 3.42377651 |
| 8 | 1422400.4976119797 | 1422397.2188858 | 3.27872617 |
| 9 | 1422422.714041921 | 1422419.3829828 | 3.33105912 |
| 10 | 1422444.9428135103 | 1422441.7900823 | 3.15273121 |
| 11 | 1422470.1321577677 | 1422466.7871534 | 3.34500436 |
| 12 | 1422510.708022163 | 1422507.4231819 | 3.28484026 |
| 13 | 1422530.6635712883 | 1422527.3203206 | 3.34325068 |
| 14 | 1422554.768864828 | 1422551.6775194 | 3.09134542 |
| 15 | 1422583.8284143084 | 1422580.6089423 | 3.219472 |
| 16 | 1422610.0763890685 | 1422606.3112673 | 3.76512176 |
| 17 | 1422634.4648094005 | 1422630.5471533 | 3.9176561 |
| 18 | 1422655.9763706902 | 1422652.8026575 | 3.17371319 |
| 19 | 1422680.902794488 | 1422677.3728405 | 3.52995398 |
| 20 | 1422702.069917121 | 1422698.803172 | 3.26674512 |
| 21 | 1422725.7541150923 | 1422722.5146106 | 3.23950449 |
| 22 | 1422751.8479511796 | 1422748.8411424 | 3.00680877 |
| 23 | 1422778.4889781792 | 1422775.0636969 | 3.42528127 |
| 24 | 1422805.358839356 | 1422801.5147692 | 3.84407015 |
| 25 | 1422850.3621374064 | 1422846.877027 | 3.4851104 |
| 26 | 1422878.1937183945 | 1422874.4412704 | 3.75244799 |
| 27 | 1422901.1909172465 | 1422897.8194259 | 3.37149134 |
| 28 | 1422922.251512224 | 1422919.0183547 | 3.23315752 |
| 29 | 1422948.6263417904 | 1422945.4080702 | 3.21827159 |
| 30 | 1422968.030615337 | 1422964.68554 | 3.34507533 |
| 31 | 1422992.3748997585 | 1422989.1106523 | 3.26424745 |
| 32 | 1423017.9092048055 | 1423014.8088045 | 3.1004003 |
| 33 | 1423045.4958179144 | 1423042.6509869 | 2.84483101 |
| 34 | 1423070.3673994318 | 1423067.1678256 | 3.19957383 |
| 35 | 1423092.543131398 | 1423089.5510855 | 2.99204589 |
| 36 | 1423114.5830711615 | 1423111.7241564 | 2.85891476 |
| 37 | 1423139.6510150507 | 1423136.5873309 | 3.06368415 |
| 38 | 1423166.5495307352 | 1423163.2952978 | 3.25423293 |
| 39 | 1423190.7180079375 | 1423188.0230103 | 2.69499763 |
| 40 | 1423217.217807731 | 1423213.9893612 | 3.22844653 |
| 41 | 1423245.66923528 | 1423242.3688976 | 3.30033768 |
| 42 | 1423266.3660336966 | 1423262.9568502 | 3.40918349 |
| 43 | 1423292.4297798679 | 1423289.3189077 | 3.11087216 |
| 44 | 1423311.859778765 | 1423308.6527554 | 3.20702336 |
| 45 | 1423331.3076682552 | 1423328.0076373 | 3.30003095 |
| 46 | 1423352.4632331615 | 1423349.3776802 | 3.08555296 |
| 47 | 1423374.8758725813 | 1423371.7601868 | 3.11568578 |
| 48 | 1423397.9585567124 | 1423394.6739521 | 3.28460461 |
| 49 | 1423422.2239879789 | 1423419.2346327 | 2.98935527 |
| 50 | 1423473.6017493969 | 1423471.0622242 | 2.53952519 |
| 51 | 1423497.8231584025 | 1423494.607834 | 3.2153244 |
| 52 | 1423561.3585337352 | 1423558.2331658 | 3.12536793 |
| 53 | 1423585.9122187826 | 1423582.3802243 | 3.53199448 |
| 54 | 1423608.401334962 | 1423604.8939339 | 3.50740106 |
| 55 | 1423633.3548261123 | 1423630.0943652 | 3.26046091 |
| 56 | 1423654.6999224501 | 1423651.0928463 | 3.60707615 |
| 57 | 1423674.8742247368 | 1423671.6035475 | 3.27067723 |
| 58 | 1423714.4180229665 | 1423711.2818172 | 3.13620576 |
| 59 | 1423738.4782008566 | 1423735.6596697 | 2.81853115 |
| 60 | 1423760.3967907052 | 1423757.6597789 | 2.7370118 |
| 61 | 1423787.5265721732 | 1423784.5529981 | 2.97357407 |
| 62 | 1423806.4071864542 | 1423803.3389402 | 3.06824625 |
| 63 | 1423827.441057177 | 1423824.6644693 | 2.77658787 |
| 64 | 1423849.2541374352 | 1423846.0671755 | 3.18696193 |
| 65 | 1423871.4133175951 | 1423868.2603809 | 3.15293669 |
| 66 | 1423893.9572173057 | 1423890.8495966 | 3.1076207 |
| 67 | 1423929.9702460445 | 1423926.7834357 | 3.18681034 |
| 68 | 1423952.6116438506 | 1423949.3790897 | 3.23255415 |
| 69 | 1423981.5309792263 | 1423978.4143989 | 3.11658032 |
| 70 | 1424005.228985842 | 1424002.2940606 | 2.93492524 |
| 71 | 1424028.065738195 | 1424024.8960224 | 3.16971579 |
| 72 | 1424050.3312942437 | 1424046.9280743 | 3.40321994 |
| 73 | 1424072.6700305904 | 1424069.3222077 | 3.34782289 |
| 74 | 1424103.3803135369 | 1424100.2731769 | 3.10713663 |
| 75 | 1424128.7276194324 | 1424125.4881913 | 3.23942813 |
| 76 | 1424163.4631411582 | 1424160.157211 | 3.30593015 |
| 77 | 1424189.5375006539 | 1424186.0949705 | 3.44253015 |
| 78 | 1424216.3668820877 | 1424213.1488562 | 3.21802588 |
| 79 | 1424239.9357175252 | 1424236.7092778 | 3.22643972 |
| 80 | 1424265.6225923258 | 1424262.2350674 | 3.38752492 |
| 81 | 1424290.084057162 | 1424286.98195 | 3.10210716 |
| 82 | 1424316.650448819 | 1424313.2963577 | 3.35409111 |
| 83 | 1424341.1229046418 | 1424338.1445663 | 2.97833834 |
| 84 | 1424366.03060094 | 1424362.9359651 | 3.09463584 |
| 85 | 1424386.186377331 | 1424383.03705 | 3.14932733 |
| 86 | 1424411.4661746626 | 1424408.6657742 | 2.80040046 |
| 87 | 1424440.0474011635 | 1424436.9807064 | 3.06669476 |
| 88 | 1424489.199262985 | 1424486.820447 | 2.37881598 |
| 89 | 1424514.5612488245 | 1424511.3351085 | 3.22614032 |
| 90 | 1424539.4828617866 | 1424535.7646296 | 3.71823218 |
| 91 | 1424562.1202274167 | 1424558.5819574 | 3.53827001 |
| 92 | 1424590.1395648336 | 1424586.4236844 | 3.71588043 |
| 93 | 1424611.7618419286 | 1424607.8820768 | 3.87976512 |
| 94 | 1424635.6252697383 | 1424632.0384701 | 3.58679963 |
| 95 | 1424667.4658902588 | 1424663.814458 | 3.65143225 |
| 96 | 1424688.629107212 | 1424685.2342062 | 3.39490101 |
| 97 | 1424711.6788923633 | 1424708.2215235 | 3.45736886 |
| 98 | 1424734.4948828272 | 1424731.0849825 | 3.40990032 |
| 99 | 1424820.6388379422 | 1424817.2803406 | 3.35849734 |
| 100 | 1424840.8056584087 | 1424837.7587966 | 3.0468618 |
| **Average = 3.242282335** | | | |
