# Supplementary material for: High spatial and temporal resolution cerebrovascular reactivity for humans and large mammals: A technological description of integrated fNIRS and niABP mapping system
Source: Front Physiol. 2023 Jan 23;14:1124268. doi: 10.3389/fphys.2023.1124268 (PMC9899997; doi:10.3389/fphys.2023.1124268)
Supplement: Supplementary file 2 [file Table2.docx]

**Appendix B – Finapres Signal Delay Tests (Multiple Runs):**

| **Test** | **End Time (seconds)** | **Start Time (seconds)** | **Time Difference (seconds)** |
| --- | --- | --- | --- |
| **Run #1 (Avg = 3.171922443)** | | | |
| 1 | 1744984.7578494416 | 1744981.8862066 | 2.87164284 |
| 2 | 1745003.5053767422 | 1744999.0934362 | 4.41194054 |
| 3 | 1745022.7394202189 | 1745019.3424149 | 3.39700531 |
| 4 | 1745048.7039333673 | 1745045.6462849 | 3.05764846 |
| 5 | 1745071.2907922731 | 1745068.1277338 | 3.16305847 |
| 6 | 1745095.7727382048 | 1745092.772115 | 3.0006232 |
| 7 | 1745122.1882954848 | 1745119.3570869 | 2.83120858 |
| 8 | 1745145.2500310054 | 1745142.1835477 | 3.0664833 |
| 9 | 1745195.278959159 | 1745192.1499013 | 3.12905785 |
| 10 | 1745235.6239941854 | 1745232.8334383 | 2.79055588 |
| **Run #2 (Avg = 2.926636219)** | | | |
| 11 | 1745685.36043424 | 1745682.483684 | 2.87675024 |
| 12 | 1745712.1883836912 | 1745709.4350764 | 2.75330729 |
| 13 | 1745745.8991035034 | 1745743.0915996 | 2.8075039 |
| 14 | 1745768.436242412 | 1745765.5621216 | 2.87412081 |
| 15 | 1745798.0278188093 | 1745794.993606 | 3.0342128 |
| 16 | 1745821.9513936203 | 1745818.9751016 | 2.97629202 |
| 17 | 1745847.4028426786 | 1745844.4679679 | 2.93487477 |
| 18 | 1745870.3599324161 | 1745867.1408778 | 3.21905461 |
| 19 | 1745891.9459685527 | 1745889.0686982 | 2.87727035 |
| 20 | 1745916.9100542027 | 1745913.9970788 | 2.9129754 |
| **Run #3 (Avg = 3.137663718)** | | | |
| 21 | 1746192.188315186 | 1746189.0625719 | 3.12574328 |
| 22 | 1746216.794587002 | 1746213.7644474 | 3.0301396 |
| 23 | 1746253.3158432334 | 1746249.7678899 | 3.54795333 |
| 24 | 1746277.780784498 | 1746274.7725504 | 3.00823409 |
| 25 | 1746300.2664056993 | 1746297.382964 | 2.88344169 |
| 26 | 1746325.1257688552 | 1746322.040646 | 3.08512285 |
| 27 | 1746346.14255815 | 1746342.9377133 | 3.20484485 |
| 28 | 1746367.778685142 | 1746364.6048845 | 3.17380064 |
| 29 | 1746388.4317451944 | 1746385.1367817 | 3.29496349 |
| 30 | 1746412.904270562 | 1746409.8818772 | 3.02239336 |
| **Run #4 (Avg = 3.172134997)** | | | |
| 31 | 1753063.632420536 | 1753060.1373952 | 3.49502533 |
| 32 | 1753089.2682495082 | 1753086.1521917 | 3.1160578 |
| 33 | 1753111.6420156015 | 1753108.4218331 | 3.2201825 |
| 34 | 1753133.8090989415 | 1753130.6135304 | 3.19556854 |
| 35 | 1753158.8147942005 | 1753155.5082042 | 3.30659 |
| 36 | 1753180.2104145696 | 1753177.0999324 | 3.11048216 |
| 37 | 1753207.6656151656 | 1753204.5503825 | 3.11523266 |
| 38 | 1753238.3033783915 | 1753235.2131344 | 3.09024399 |
| 39 | 1753266.8206483067 | 1753263.7721577 | 3.0484906 |
| 40 | 1753286.8119780996 | 1753283.7885017 | 3.02347639 |
| **Run #5 (Avg = 3.153024524)** | | | |
| 41 | 1753439.1755917172 | 1753436.0227337 | 3.15285801 |
| 42 | 1753459.6272660436 | 1753456.4204643 | 3.20680174 |
| 43 | 1753484.9084568836 | 1753481.6661027 | 3.24235418 |
| 44 | 1753513.201084157 | 1753509.9650278 | 3.23605635 |
| 45 | 1753540.051397654 | 1753536.868023 | 3.18337465 |
| 46 | 1753565.3804666437 | 1753562.1212381 | 3.25922854 |
| 47 | 1753590.5190636374 | 1753587.3058848 | 3.21317883 |
| 48 | 1753613.6148063047 | 1753610.8391725 | 2.7756338 |
| 49 | 1753640.0593160943 | 1753637.1367334 | 2.92258269 |
| 50 | 1753665.0337929535 | 1753661.6956165 | 3.33817645 |
| **Run #6 (Avg = 3.245329524)** | | | |
| 51 | 1753772.0905647136 | 1753768.7413075 | 3.34925721 |
| 52 | 1753799.1803049408 | 1753795.9683131 | 3.21199184 |
| 53 | 1753822.790988897 | 1753819.4029945 | 3.38799439 |
| 54 | 1753844.7255290993 | 1753841.8792613 | 2.84626779 |
| 55 | 1753865.621397955 | 1753862.4309333 | 3.19046465 |
| 56 | 1753888.8224596144 | 1753885.2966456 | 3.52581401 |
| 57 | 1753937.2750087925 | 1753934.1889031 | 3.08610569 |
| 58 | 1753966.7927676998 | 1753963.4707529 | 3.32201479 |
| 59 | 1753994.5214846001 | 1753991.2950771 | 3.2264075 |
| 60 | 1754020.34570387 | 1754017.0387265 | 3.30697737 |
| **Run #7 (Avg = 3.140618866)** | | | |
| 61 | 1754131.9401100732 | 1754128.8959977 | 3.04411237 |
| 62 | 1754158.5813973036 | 1754155.2945485 | 3.2868488 |
| 63 | 1754183.5625570375 | 1754180.4219563 | 3.14060073 |
| 64 | 1754210.564196519 | 1754207.5790936 | 2.98510291 |
| 65 | 1754232.9895181663 | 1754229.8283116 | 3.16120656 |
| 66 | 1754257.0974213667 | 1754254.2454237 | 2.85199766 |
| 67 | 1754285.1613034252 | 1754281.8526209 | 3.30868252 |
| 68 | 1754307.1425467592 | 1754304.0384164 | 3.10413035 |
| 69 | 1754330.0386051196 | 1754326.6992486 | 3.33935651 |
| 70 | 1754351.0903254545 | 1754347.9061752 | 3.18415025 |
| **Run #8 (Avg = 3.137570351)** | | | |
| 71 | 1754523.4351174433 | 1754520.5231372 | 2.91198024 |
| 72 | 1754546.4806792657 | 1754543.1999673 | 3.28071196 |
| 73 | 1754570.4140600006 | 1754567.154409 | 3.259651 |
| 74 | 1754593.6846558282 | 1754590.5943385 | 3.09031732 |
| 75 | 1754617.9802658183 | 1754614.5307191 | 3.44954671 |
| 76 | 1754641.9658728673 | 1754638.7928333 | 3.17303956 |
| 77 | 1754666.4512325455 | 1754663.4034493 | 3.04778324 |
| 78 | 1754688.6075222364 | 1754685.7073044 | 2.90021783 |
| 79 | 1754716.3924352885 | 1754713.3635321 | 3.02890318 |
| 80 | 1754744.9730587748 | 1754741.7395063 | 3.23355247 |
| **Run #9 (Avg = 3.748480214)** | | | |
| 81 | 1754839.491829456 | 1754836.302739 | 3.18909045 |
| 82 | 1754866.5115704648 | 1754863.6290994 | 2.88247106 |
| 83 | 1754896.5071138863 | 1754893.2668561 | 3.24025778 |
| 84 | 1754926.5327882497 | 1754923.5878017 | 2.94498654 |
| 85 | 1754950.550816143 | 1754947.3351474 | 3.21566874 |
| 86 | 1754975.007018732 | 1754972.2375041 | 2.76951463 |
| 87 | 1755002.9301502563 | 1754998.6271704 | 4.30297985 |
| 88 | 1755027.4782792085 | 1755022.4115724 | 5.0667068 |
| 89 | 1755049.9571590908 | 1755044.7271486 | 5.23001049 |
| 90 | 1755072.441087902 | 1755067.7979721 | 4.6431158 |
| **Run #10 (Avg = 2.999826926)** | | | |
| 91 | 1755186.321968869 | 1755183.3187483 | 3.00322056 |
| 92 | 1755214.1673835109 | 1755211.0572179 | 3.11016561 |
| 93 | 1755236.7639023499 | 1755233.7691626 | 2.99473974 |
| 94 | 1755257.6439224144 | 1755254.4133424 | 3.23058001 |
| 95 | 1755282.8346128096 | 1755279.6327589 | 3.2018539 |
| 96 | 1755307.2809842548 | 1755304.1445298 | 3.13645445 |
| 97 | 1755328.850841493 | 1755325.8509035 | 2.99993799 |
| 98 | 1755351.841101343 | 1755348.7245431 | 3.11655824 |
| 99 | 1755379.2012844565 | 1755375.9041759 | 3.29710855 |
| 100 | 1755400.645499117 | 1755398.7378489 | 1.90765021 |
| **Average of 10 Runs = 3.183320778** | | | |
